# Draft Genome Sequence of *Cobetia* sp. UCD-24C, Isolated from Roots and Leaves of the Seagrass *Zostera marina*

**DOI:** 10.1128/genomeA.00116-16

**Published:** 2016-03-10

**Authors:** Alexandra Alexiev, Megan L. Krusor, Guillaume Jospin, Jenna M. Lang, Jonathan A. Eisen, David A. Coil

**Affiliations:** a Davis Genome Center, University of California Davis, Davis, California, USA; b Department of Earth and Planetary Sciences, University of California Davis, Davis, California, USA; c Departments of Evolution and Ecology and Medical Microbiology and Immunology, University of California Davis, Davis, California, USA

## Abstract

Here, we present the 4,230,758-bp draft genome for *Cobetia* sp. UCD-24C. This strain was isolated from *Zostera marina* roots collected in Woods Hole, Massachusetts, USA.

## GENOME ANNOUNCEMENT

The *Cobetia* genus was suggested by Arahal et al. and is most closely related to *Halomonas* and *Chromohalobacter*. The type strain was described as aerobic, Gram-negative, motile, and with rod-shaped cells that occur singly or in pairs. It is slightly halophilic and requires salt for growth (1). When grown in a mixed culture with two other marine bacteria, some *Cobetia* spp. can produce a bioflocculant that removes turbidity and reduces chemical oxygen demand, resulting in its suggested use for wastewater treatment (2). *Cobetia* UCD-24C was isolated from seagrass (*Zostera marina*) roots collected from plants in Woods Hole, Massachusetts, USA (41°31′30.0″N 70°40′22.9″W). This sampling and culturing project was done as part of a collaboration between researchers at the University of California, Davis, and the University of Oregon called the Seagrass Microbiome Project (http://www.seagrassmicrobiome.org). The project seeks to characterize and analyze the microbial communities living in and on seagrasses.

Bacterial isolates were grown and double dilution struck on Luria broth agar (Difco), seawater agar, 10% diluted seawater agar, and *Azotobacter* isolation medium agar. Isolates were incubated at 25°C for 1 to 21 days. Scrapings were then frozen in 25% glycerol for long-term storage. The isolates were later thawed and grown in seawater nutrient agar medium (ATCC medium 2205, using InstantOcean instead of synthetic seawater). DNA was subsequently extracted using a Wizard Genomic DNA purification kit (Promega) from a fresh overnight culture.

A paired-end library was produced using a Nextera DNA sample prep kit (Illumina) and sequenced on an Illumina HiSeq platform. Sequencing resulted in 1,355,001 reads with a read length of 250 bp and approximately 160× coverage. The genome size is 4,230,758 bp, and the GC content was 62.5%. Sequences were processed by the A5-miseq assembly pipeline (3, 4), which automates error correction, data cleaning, contig assembly, and quality control. The completeness of the genome was assessed using PhyloSift (5), which utilizes a list of 37 highly conserved, single-copy marker genes (6). One copy of each marker gene was found in the assembly. Automated annotation was done using the RAST annotation server (7). A BLAST search and phylogenetic analysis using the assembled full-length 16S rRNA gene identified the isolate as a species of *Cobetia* (tree available at https://dx.doi.org/10.6084/m9.figshare.2066541.v1). This tree is ambiguous with respect to determination of species, and therefore we have only given a strain designation.

### Nucleotide sequence accession numbers.

This genome sequence has been deposited at DDBJ/EMBL/GenBank under the accession number LJTD00000000. The version described in this paper is LJTD00000000.1.
